# [Corrigendum] Adjudin delays cellular senescence through Sirt3-mediated attenuation of ROS production

**DOI:** 10.3892/ijmm.2026.6001

**Published:** 2026-09-23

**Authors:** Keyi Geng, Ningzhen Fu, Xiao Yang, Weiliang Xia

Int J Mol Med 42: 3522-3529, 2018; DOI: 10.3892/ijmm.2018.3917

Following the publication of the above article, a concerned reader drew to the authors' attention that, regarding the images of the mouse embryo fibroblast (MEF) cells with senescence-associated β-galactosidase (SA-β-gal) staining shown in Fig. 1B on p. 3254, the panels on the far left and the far right on the bottom row of data [showing treatments with 0 *μ*M and 40 *μ*M adjudin respectively in the presence of hydroxyurea (HU)] contained an overlapping section of data (albeit after turning the right-hand panel through 90°), such that data which were intended to show the results from differently performed experiments had apparently been derived from the same original source. In addition, the reader also observed that one of the antibodies the authors had used in their study was apparently selected inappropriately: The antibody (cat. no. ab51243) that the authors reported to have used is specific for the protein known as p16-ARC (or ARPC5), not the apoptosis-associated protein p16-INK4a, as was intended.

After re-examining their original data, the authors have realized that the data in Fig. 1B were assembled incorrectly. A revised version of Fig. 1 is shown on the next page, now featuring data for all the panels in Fig. 1B from one of the repeated sets of experiments. Concerning the potential selection of an inappropriate antibody to probe for p16 protein, the authors expressed their regret that they had not retained their original receipt for the purchase of this antibody, and were not in a position to prove whether or not the antibody had been chosen legitimately for the affected experiments; however, an increased abundance of p16-INK4a was used as an indicator of cellular senescence in combination with the abundance of p21 and the fraction of cells that contained a SA-β-gal signal. In view of this, the authors considered that removing the p16 data from the paper would not compromise the conclusions drawn in this study.

After considering this appeal, the Editor has approved the authors' request that the p16 data be omitted from this study. Therefore, all references to 'p16' in this study (as were featured in the Abstract, and the Materials and methods, Results and Discussion sections) should be considered to be eliminated from this paper; the revised version of Fig. 1 that has been provided to take account of the new data in Fig. 1B also no longer includes the p16 data, and a new version of Fig. 3 has also been provided (on the third page of this corrigendum), again with the p16 data excluded.

Note that the revisions made to these figures, and the omission of the data relating to p16, do not affect the overall conclusions reported in the paper. The authors express their gratitude to the Editor of *International Journal of Molecular Medicine* for allowing them the opportunity to publish this corrigendum, and apologize to the readership for any inconvenience caused.

## Figures and Tables

**Figure 1:**
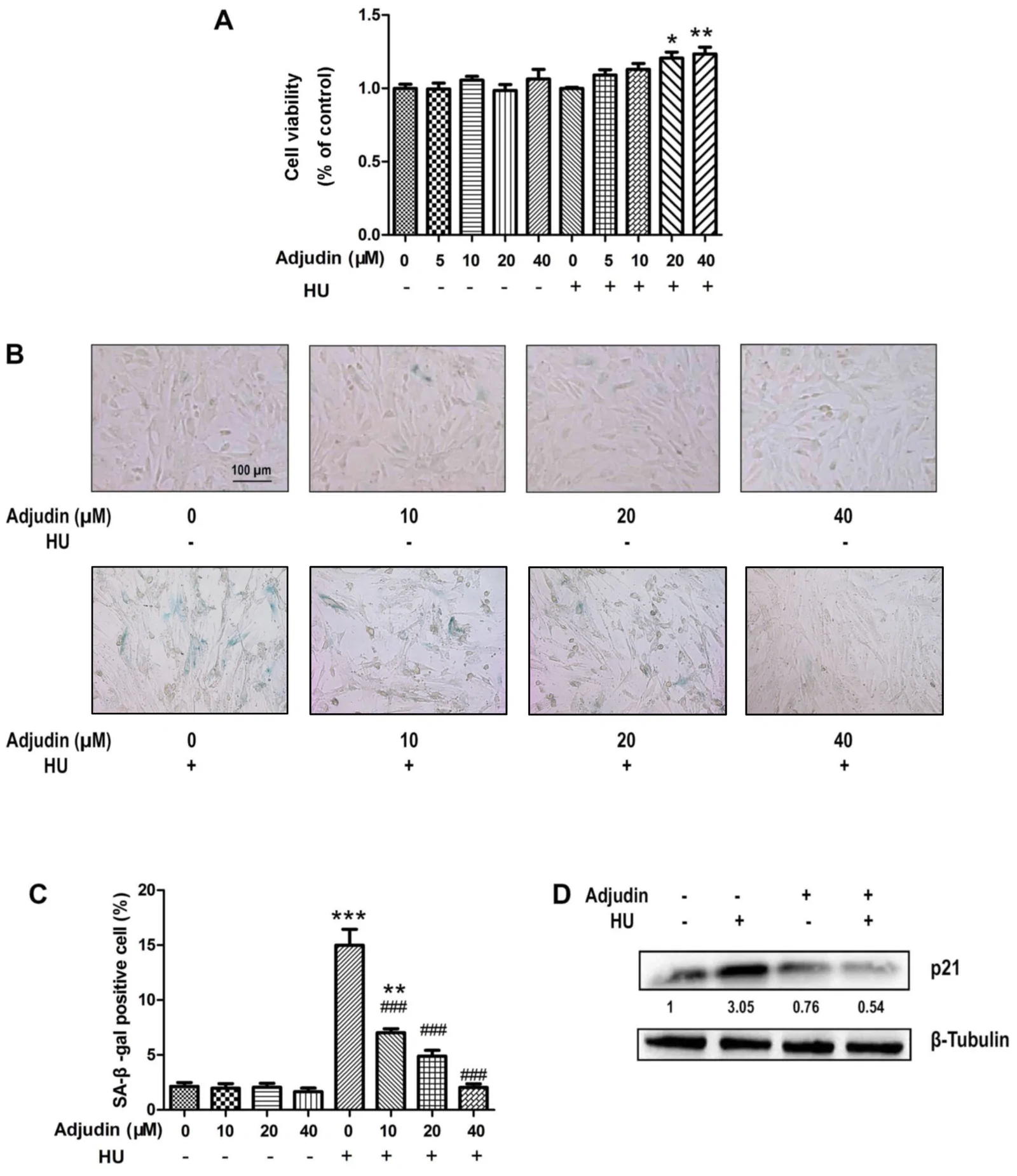
Adjudin attenuates HU-induced cell senescence. MEF cells were pretreated with indicated concentrations of adjudin for 1 h and then stimulated with 6 mM HU for a 24-h incubation period. (A) MEF cells were pretreated with adjudin at 5, 10, 20 and 40 *μ*M in the absence or presence of HU and cell viability was determined by the cell counting kit-8 assay. (B) MEF cells were treated with adjudin at 10, 20 and 40 *μ*M in the absence or presence of HU and stained for SA-β-gal. Scale bar, 100 *μ*m. (C) Percentage of SA-β-gal activity positive cells was determined by counting blue cells counting 400 cells/well. (D) Cells were pretreated with adjudin at 40 *μ*M. Cell lysates were analyzed by immunoblotting with antibodies specific to p21. β-tubulin was used as a reference. Data are presented as the mean ± standard deviation of three independent experiments. ^*^P<0.05, ^**^P<0.01 and ^***^P<0.001 vs. control treatment; ^###^P<0.001 vs. HU alone treatment. HU, hydroxyurea; MEF, mouse embryo fibroblast; SA-β-gal, senescence-associated β-galactosidase.

**Figure 3:**
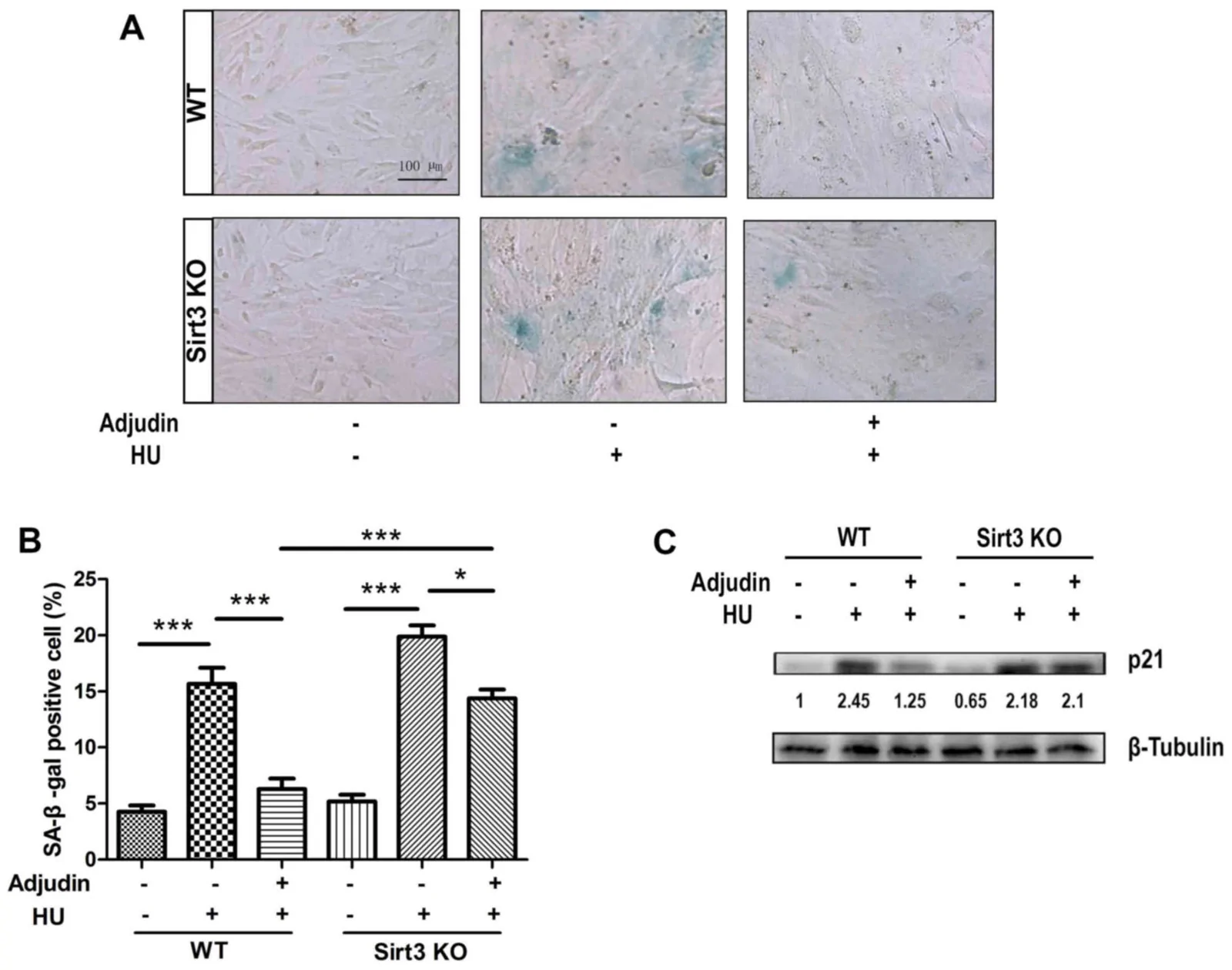
Anti-aging effect of adjudin is mediated by Sirt3. Cells were pretreated with 40 *μ*M of adjudin for 1 h and then stimulated with 6 mM HU for a 24-h incubation period. (A) Cells were stained for SA-β-gal. Scale bar, 100 *μ*m. (B) Percentage of SA-β-gal activity positive cells was determined by counting 400 cells/well. (C) Cell lysates were analyzed by immunoblotting with antibodies specific to p21. β-tubulin was used as a reference for equal loading. Data are presented as the mean ± standard of three independent experiments. ^*^P<0.05 and ^***^P<0.001. WT, wild-type; Sirt3, sirtuin; KO knockout; HU, hydroxyurea.

